# Gene shaving using a sensitivity analysis of kernel based machine learning approach, with applications to cancer data

**DOI:** 10.1371/journal.pone.0217027

**Published:** 2019-05-23

**Authors:** Md. Ashad Alam, Mohammd Shahjaman, Md. Ferdush Rahman, Fokhrul Hossain, Hong-Wen Deng

**Affiliations:** 1 Tulane Center of Bioinformatics and Genomics, Department of Global Biostatistics and Data Science, Tulane University, New Orleans, LA 70112, United States of America; 2 Department of Statistics, Hajee Mohammad Danesh Science and Technology University, Dinajpur 5200, Bangladesh; 3 Department of Statistics, Begum Rokeya University, Rangpur 5400, Bangladesh; 4 Department of Marketing, Begum Rokeya University, Rangpur 5400, Bangladesh; 5 Department of Genetics, Stanley S. Scott Cancer Center, LSU Health Sciences Center, Louisiana State University, New Orleans, LA 70112, United States of America; Instituto Nacional de Medicina Genomica, MEXICO

## Abstract

**Background:**

Gene shaving (GS) is an essential and challenging tools for biomedical researchers due to the large number of genes in human genome and the complex nature of biological networks. Most GS methods are not applicable to non-linear and multi-view data sets. While the kernel based methods can overcome these problems, a well-founded positive definite kernel based GS method has yet to be proposed for biomedical data analysis.

**Methods and findings:**

Since the kernel based methods on genomic information can improve the prediction of diseases, here we proposed a noble method, “kernel based gene shaving” which is based on the influence function of kernel canonical correlation analysis. To investigate the performance of the proposed method in comparison to state-of-the-art-method in gene saving, we analyzed extensive simulated and real microarray gene expression data set. The performance metrics including true positive rate, true negative rate, false positive rate, false negative rate, misclassification error rate, the false discovery rate and area under curves were computed for each methods. In colon cancer data analysis, the proposed method identified a significant subsets of 210 genes out of 2000 genes and suggestive superior performance compared with other methods. The proposed method can be applied to the study of other disease process where two view data is a common task.

**Conclusions:**

We addressed the challenge of finding unique kernel based GS methods by using the influence function of kernel canonical correlation analysis. The proposed method has shown to have better performance than state-of-the-art-methods in gene saving and has identified many more significant gene interactions, suggesting that genes function in a concerted effort in colon cancer. In similar biomedical data analysis, kernel based methods could be applied to select a potential subset of genes. The positive definite kernel based methods can overcome the non-linearity problem and improve the prediction process.

## Introduction

Gene shaving (GS), to identify significant subsets of the genes, is an important research area in the analysis of DNA microarray gene expression data for biomedical discovery. GS methods aim to remove redundant and irrelevant genes so that performing in supervised learning will be more accurate [1, 2]. It leads to gene discovery relevant for a particular target annotation and contributes to better medical diagnosis and prognosis. GS is not relevant to the hierarchical clustering and other widely used methods for analyzing gene expression in the genome-wide association studies. GS leads to gene discovery relevant for a specific target annotation. The selected genes using GS play an important role in the gene expression data analysis since they can differentiate samples from different populations [3–6]. Despite their successes, these studies are often hampered by their relatively low reproducibility, nonlinearity and multi-view data.

The incorporation of various statistical machine learning approaches into genomic analysis is a rather recent area of study. Since large-scale microarray data presents significant challenges for the statistical data analysis, in addition the classical approaches, there is a need for an advanced method. The kernel methods (methods based on positive definite kernel) are the appropriate tools to deal with such data set that map data from a high dimensional space to a feature space using a nonlinear feature map. The main advantage of these methods is to combine statistics and geometry in an effective way [7–9]. As a machine learning approach, kernel canonical correlation analysis (kernel CCA) have been extensively studied for decades to analyze multi-view data set [10–12]. Using the influence function (IF) of kernel canonical correlation analysis, we proposed a novel kernel method to select a significant subset of genes of biomedical data analysis.

Nowadays, IF based methods (e.g., sensitivity analysis) have been used to detect an influence observation. IF is used to find a set of vectors that have much greater effect on the estimator of the parameter [13]. A visualization method for detecting influential observations using the IF of Kernel principal component analysis has been proposed by Debruyne et al. [14]. Filzmoser et al. also developed a method for outlier identification in high dimensions [15]. However, these methods are limited to a single view data set. Due to the properties of eigen-decomposition, kernel CCA and its variant are still well used methods for the biomedical data analysis [16–18].

The contribution of this paper is three-fold. First, we address the IF of kernel CCA. Second, we use the distribution based methods to confirm the influential observations. Finally, the proposed method is applied to identify a set of genes in both synthesized and gene expression data. The accuracy of the proposed method shows superior performance compared to the the state-of- the-art-method in gene saving based on the area under curves (AUC). In colon cancer data analysis, we used the proposed method to identify genes and perform pathway analysis [the gene ontology (GO) of biological process categories, Kyoto Encyclopedia of Genes and Genomes (KEGG)] and gene-gene interaction networks. We found that identified genes function in a concerted effort and have biological relevance to colon cancer. In addition, the selected genes based classification is superior than selected genes by other methods as well as classification using all genes. For any biomedical data analysis, the proposed method could be applied to select a potential subset of genes.

The remainder of the paper is organized as follows. In the materials and methods section, we provide a brief review of positive definite kernel, kernel CCA and IF of kernel CCA. The utility of the proposed method is demonstrated by both simulated and real data analysis from an colon cancer study in the experimental results section. In the discussion section, we also summarize our findings and give a perspective for future research.

## Materials and methods

### Positive definite kernel

In kernel methods, a nonlinear feature map is defined by positive definite kernel. It is known that a positive definite kernel *k* is associated with a Hilbert space H, called reproducing kernel Hilbert space (RKHS), consisting of functions on X so that the function value is reproduced by the kernel [19]. For any function f∈H and a point X∈X, the function value *f*(*X*) is $f(X)={\langle f(\cdot ),k(\cdot ,X)\rangle }_{H},$ where ${\langle ,\rangle }_{H}$ in the inner product of H is called the reproducing property. Replacing *f* with $k(\cdot ,\tilde{X})$ yields $k(X,\tilde{X})={\langle k(\cdot ,X),k(\cdot ,\tilde{X})\rangle }_{H}$ for any $X,\tilde{X}\in X$. A symmetric kernel *k*(⋅, ⋅) defined on a space X is called positive definite, if for an arbitrary number of points $X_{1},X_{2}\ldots ,X_{n}\in X$ the Gram matrix (*k*(*X*_*i*_, *Y*_*j*_))_*ij*_ is positive semi-definite. To transform data for extracting nonlinear features, the mapping Φ:X→H is defined as **Φ**(*X*) = *k*(⋅, *X*), which is a function of the first argument. This map is called the f feature map, and the vector **Φ**(*X*) in H is called the feature vector. The inner product of two feature vectors is then ${\langle \Phi \left(X\right),\Phi \left(\tilde{X}\right)\rangle }_{H}=k\left(X,\tilde{X}\right).$ This is known as the kernel trick. By this trick the kernel can evaluate the inner product of any two feature vectors efficiently without knowing an explicit form of **Φ**(⋅) [7–9].

### Kernel canonical correlation analysis

Kernel CCA has been proposed as a nonlinear extension of linear CCA [10]. Researchers have extended the standard kernel CCA with an efficient computational algorithm [20]. Over the last decade, kernel CCA has been used for various tasks [21–23]. Given two sets of random variables *X* and *Y* with two functions in the RKHS, $f_{X}\left(\cdot \right)\in H_{X}$ and $f_{Y}\left(\cdot \right)\in H_{Y}$, the optimization problem of the random variables *f*_*X*_(*X*) and *f*_*Y*_(*Y*) is

$$\rho =\underset{\begin{array}{c} f_{X}\in H_{X},f_{Y}\in H_{Y}\\ f_{X}\neq 0,\;f_{Y}\neq 0 \end{array}}{\text{max}}\text{Corr}\left(f_{X}\left(X\right),f_{Y}\left(Y\right)\right).$$ (1)

The optimizing functions *f*_*X*_(⋅) and *f*_*Y*_(⋅) are determined up to scale.

Using a finite sample, we are able to estimate the desired functions. Given an i.i.d sample, ${\left(X_{i},Y_{i}\right)}_{i=1}^{n}$ from a joint distribution *F*_*XY*_, by taking the inner product with elements or “parameters” in the RKHS, we have features $f_{X}\left(\cdot \right)={\langle f_{X},\Phi_{X}\left(X\right)\rangle }_{H_{X}}=\sum_{i=1}^{n}a_{X}^{i}k_{X}\left(\cdot ,X_{i}\right)$ and $f_{Y}\left(\cdot \right)={\langle f_{Y},\phi_{Y}\left(Y\right)\rangle }_{H_{Y}}=\sum_{i=1}^{n}a_{Y}^{i}k_{Y}\left(\cdot ,Y_{i}\right)$, where *k*_*X*_(⋅, *X*) and *k*_*Y*_(⋅, *Y*) are the associated kernel functions for $H_{X}$ and $H_{Y}$, respectively. The kernel Gram matrices are defined as $K_{X}≔{\left(k_{X}\left(X_{i},X_{j}\right)\right)}_{i,j=1}^{n}$ and $K_{Y}≔{\left(k_{Y}\left(Y_{i},Y_{j}\right)\right)}_{i,j=1}^{n}$. We need the centered kernel Gram matrices **M**_*X*_ = **CK**_*X*_**C** and **M**_*Y*_ = **CK**_*Y*_**C**, where $C=I_{n}-\frac{1}{n}B_{n}$ with $B_{n}=1_{n}1_{n}^{T}$ and **1**_*n*_ is the vector with *n* ones. The empirical estimate of Eq (1) is then given by

$$\hat{\rho }=\underset{\begin{array}{c} f_{X}\in H_{X},f_{Y}\in H_{Y}\\ f_{X}\neq 0,\;f_{Y}\neq 0 \end{array}}{\text{max}}\frac{\hat{\text{Cov}}\left(f_{X}\left(X\right),f_{Y}\left(Y\right)\right)}{{\left[\hat{\text{Var}}\left(f_{X}\left(X\right)\right)\right]}^{1/2}{\left[\hat{\text{Var}}\left(f_{Y}\left(Y\right)\right)\right]}^{1/2}},$$

where

$$\begin{array}{c} \hat{\text{Cov}}\left(f_{X}\left(X\right),f_{Y}\left(Y\right)\right)=\frac{1}{n}a_{X}^{T}M_{X}M_{Y}a_{Y}\\ \hat{\text{Var}}\left(f_{X}\left(X\right)\right)=\frac{1}{n}a_{X}^{T}M_{X}^{2}a_{X}\;\\ \hat{\text{Var}}\left(f_{Y}\left(Y\right)\right)=\frac{1}{n}a_{Y}^{T}M_{Y}^{2}a_{Y}, \end{array}$$

where **a**_*X*_ and **a**_*Y*_ are the directions of *X* and *Y*, respectively.

### Influence function of the kernel canonical correlation analysis

Since 1974, the IF plays an important role for detecting outlying multivariate observations in statistical analysis. The IF can usually be defined on first order approximation for estimators of parameters in a multivariate population which indicates where in the n-dimensional space of observations. The observed vectors should have a large effect on the value of the estimator of the parameter. For a sample of observation vectors, we can define the IF based on empirical distribution (EIF) to find set of these vectors that have much greater effect on the estimator. This vector is called set of outline vector [13]. In many situation outliers are often the special point of interest and their recognition is the main goal of the investigation. Although, there are several approaches to identify outliers in multivariate data analysis. The goal of this paper is to identify a set of outline observations for two view data set using IF of kernel CCA.

Using the idea of IF of the linear PCA, the kernel PCA, and the linear CCA, the IF of kernel CCA has been proposed by Alam et al., [18]. To define, given two sets of random variables (*X*, *Y*) having the distribution *F*_*XY*_ and the *j*-th kernel CC (*ρ*_*j*_) and kernel CVs (*f*_*jX*_(*X*) and *f*_*jX*_(*Y*)), the influence functions of kernel CC at *Z*′ = (*X*′, *Y*′) is given by

$$\text{IF}\left(Z^{\prime },\rho_{j}^{2}\right)=-\rho_{j}^{2}{\tilde{f}}_{\text{jX}}^{2}\left(X^{\prime }\right)+2\rho_{j}{\tilde{f}}_{\text{jX}}\left(X^{\prime }\right){\tilde{f}}_{\text{jY}}\left(Y^{\prime }\right)-\rho_{j}^{2}{\tilde{f}}_{\text{jY}}^{2}\left(Y^{\prime }\right),$$

where ${\tilde{f}}_{X}\left(X\right)=\langle f_{X},{\tilde{k}}_{X}\left(\cdot ,X\right)$ and ${\tilde{f}}_{Y}\left(Y\right)=\langle f_{Y},{\tilde{k}}_{X}\left(\cdot ,Y\right)$. The above theorem has been proved on the basis of previously established ones, such as the IF of linear PCA [24, 25], the IF of linear CCA [26], and the IF of kernel PCA, respectively. The details proof is given in [18].

Let ${\left(X_{i},Y_{i}\right)}_{i=1}^{n}$ be a sample from the empirical joint distribution *F*_*nXY*_. The EIF of kernel CC at (*X*′, *Y*′) for all points (*X*_*i*_, *Y*_*i*_) is defined as

$$\text{EIF}\left(X_{i},Y_{i},X^{\prime },Y^{\prime },\rho_{j}^{2}\right)=\hat{\text{IF}}\left(X^{\prime },Y^{\prime },{\hat{\rho }}_{j}^{2}\right)=-{\hat{\rho }}_{j}^{2}{\hat{\tilde{f}}}_{\text{jX}}^{2}\left(X^{\prime }\right)+2{\hat{\rho }}_{j}^{2}{\hat{\tilde{f}}}_{\text{jX}}\left(X^{\prime }\right){\hat{\tilde{f}}}_{\text{jY}}\left(Y^{\prime }\right)-{\hat{\rho }}_{j}^{2}{\hat{\tilde{f}}}_{\text{jY}}^{2}\left(Y^{\prime }\right)$$ (2)

Using the above result, we can identify a set of observations based on its influence values. To demonstrate, we proposed a noble method, with application to DNA microarray gene expression data. This novel method can be applied to the study any disease processes, where two-view data analysis is a common task. The proposed approach consists of two basic parts: a step that aims to calculate influence value of each gene and a step that aims to determine the outline gene. For the first step, we use EIF in Eq (2) and we can use a any univariate outliers detection tools. To extract the outliers of the genes, we have considered distribution based tools.

### Kernel choice

In kernel based learning, choosing a suitable kernel is key for favorable results. Most of unsupervised kernel methods suffer from the problem of kernel choice. The liner kernel is just used the underlying Euclidean space to define the similarity measure. Whenever the dimensionality of the input space, **X** is very high, this might allow for more complexity in the function class than what we could measure and assess otherwise. It has limitation of linearity. Using a polynomial kernel it is possible to use the higher order correlation between the data in the different purposes. But, due to the finite bounded degree such kernel will not provide us with guarantees for a good dependency measure. In addition both liner and polynomial kernels are non-robust.

The Gaussian kernel, is a radial basis function kernels that maps **X** into an infinite dimensional space. The Gaussian kernel is defined as:

$$k_{G}\left(X,\tilde{X}\right)=e^{\frac{1}{2\sigma^{2}}-||X-\tilde{X}{\left|\right|}^{2}},\;\left(\sigma >0\right).$$

This most applicable kernel in kernel methods has a number of theoretical properties (e.g., boundedness, consistent, characteristic, universality, robustness etc.) [27]. In this paper we consider the Gaussian kernel and use the median of the pairwise distance as a bandwidth [28, 29].

The assumption of kernel methods (methods based on positive definite kernel) is that the data should be a non-empty set. The kernel methods are independent of the dimensions. Its allow us to construct spaces of functions on an arbitrary set with the appropriate structure of a Hilbert space. By the reproducing property, computing the inner product on RKHS is easy and the computational cost only depends on the sample size. It is true that kernel methods may have computational issues for very large data set in handling Gram matrices of sample size. However, recent developments on approximation methods such as random Fourier features enables us to apply kernel methods to data size of millions.

### Relevant approaches

While the proposed approach is designed for two view data set, we compare its performance against other relevant algorithms in univariate data or multivariate data (one view data) set only, since a two view data comparison is not feasible. To demonstrate the performance of the proposed method in a comparison, we examine four popular gene selection methods: T-test, significance analysis of microarrays (SAM), Linear Models for Microarray and RNA-Seq Data (LIMMA) and principal components to identify outliers (PCout) [15, 30–32]. Computing a t-test statistic can be problematic because the variance estimates can be skewed by genes having a very low variance [30]. For each gene, SAM gives a score on the basis of change in gene expression relative to the standard deviation of repeated measurements. For genes with scores greater than an adjustable threshold, SAM uses permutations of the repeated measurements to estimate the percentage of genes identified by chance, the false discovery rate (FDR) [31]. LIMMA contains rich features for handling complex experimental designs and for information borrowing to overcome the problem of small sample sizes. This linear modelling strategy (beyond the intended analysis of gene expression data) has been found to have many applications [32]. A computationally fast procedure for identifying outliers is presented that is particularly effective in high dimensions. This algorithm not only utilizes simple properties in the transformed space but also needs less computational time than existing methods for outliers detection, and is suitable for use on very large data sets [15]. But it has limitation of linearity and a single view data set. We used all of these methods to compare to the proposed method.

## Experimental results

We have used both simulated and real microarray gene expression data set of colon cancer [33]. To compare relevant approaches (T-test, SAM, LIMMA and PCout) we used four R packages including STATS, SAMR, LIMMA and PCout, respectively. The performance measures including true positive rate (TPR), true negative rate (TNR), false positive rate (FPR), false negative rate (FNR), misclassification error rate (MER), FDR and AUC have been evaluated for each of the methods as previously described [34]. To compute the performance measures, we used R packages, which are available in the comprehensive R archive network or bioconductor.

### Simulation study

To investigate the performance of the proposed method in comparison with four popular methods as mentioned above with *k* = 2 groups, we considered gene expression profiles from both normal distribution and t-distribution. We also considered data set of both small-and-large-sample cases with different percentages of differently expressed (DE) genes.

### Simulated gene expression profiles generated from normal distribution

We used a one-way ANOVA model to generate simulated data sets from normal distribution

$$\begin{array}{c} x_{ijk}=\mu_{ik}+\epsilon_{ijk};\\ \left(i=1,2,\cdots ,G;j=1,2,\cdots ,n_{k};k=1,2,\cdots ,m\right) \end{array}$$ (3)

where *x*_*ijk*_, i is the expression of the *i*th gene for the *j*th samples in k group, *μ*_*ik*_ is the mean of all expressions of ith gene in the kth group and *ϵ*_*ijk*_ is the random error which usually follows a normal distribution with mean zero and variance *σ*^2^.

**Fig 1:**
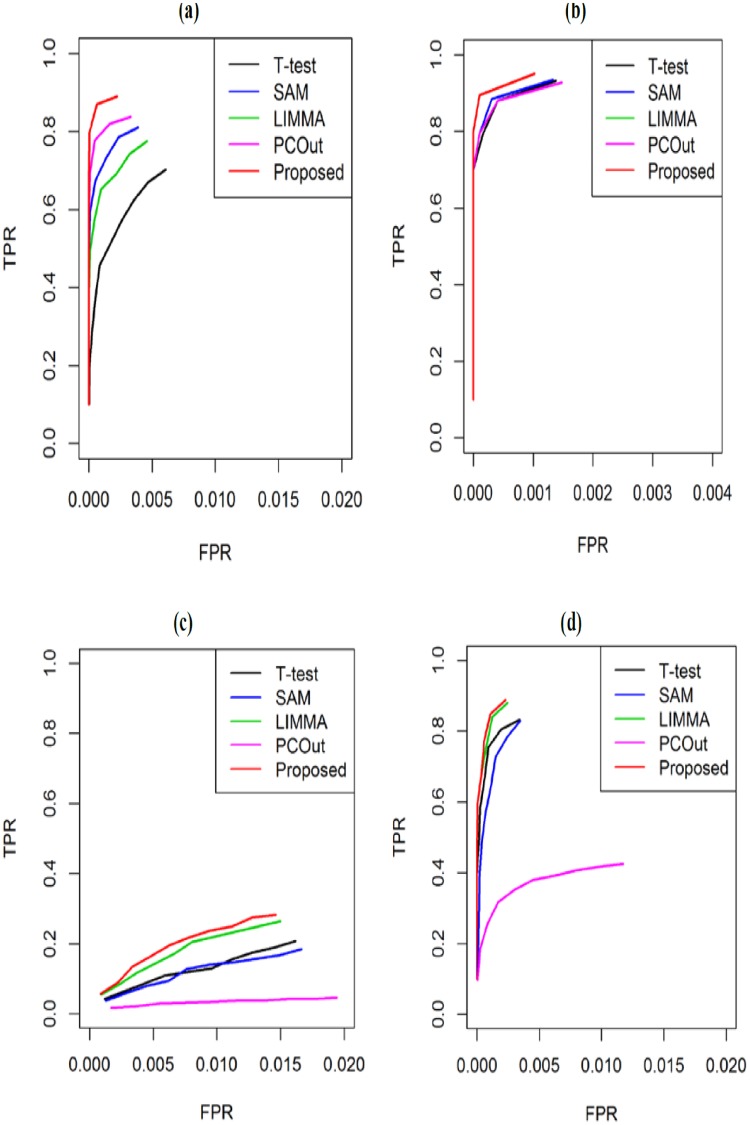
Performance evaluation using ROC-curve produced by the four methods (T-test, SAM, LIMMA, PCout and Proposed) based on 100 datasets with pDEG = 0.02. Datasets were generated from normal distribution for (a) and (b) and datasets were generated from t-distribution for (c) and (d), where (a) and (c) represents ROC curve for small-sample case (*n*_1_ = *n*_2_ = 3) and (b) and (d) represents ROC curve for large-sample case (*n*_1_ = *n*_2_ = 15).

**Fig 2:**
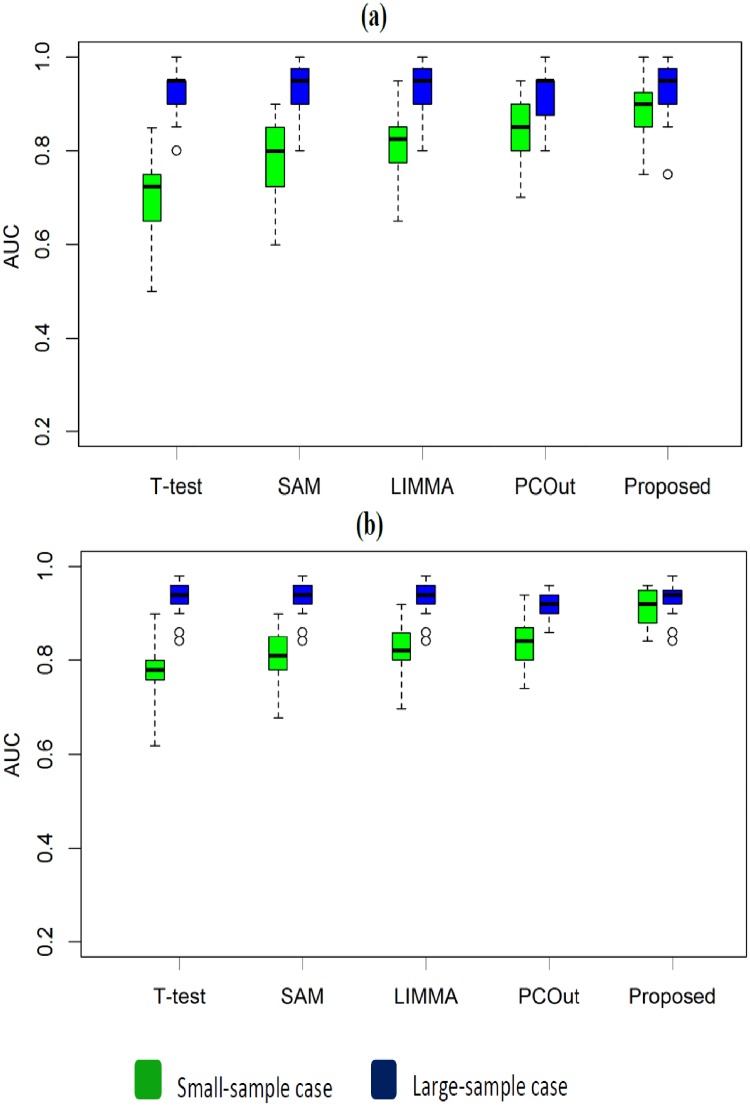
Performance evaluation using boxplot of AUC values produced by the four methods (T-test, SAM, LIMMA, PCout and Proposed) based on 100 datasets were taken from normal distribution for small-and large-sample cases (a) Boxplot of AUC values with proportion of DE gene = 0.02. (b) Boxplot of AUC values with proportion of DE gene = 0.06. Each dataset contains G = 1000 genes.

To investigate the performance of the proposed method in a comparison of other four popular methods as early mentioned for *k* = 2 groups, we generated 100 data sets using 100 times of simulations for both small (*n*_1_ = *n*_2_ = 3) and large (*n*_1_ = *n*_2_ = 15) sample cases using Eq (3). The means and the common variance of both groups were set as (*μ*_*i*1_, *μ*_*i*2_) ∈ (3, 5) and *σ*^2^ = 0.1, accordingly. Each data set for each case represented the gene expression profiles of *G* = 1000 genes, with *n* = (*n*_1_+ *n*_2_) samples. The proportions of DE gene (pDEG) were set to 0.02 and 0.06 for each of the 100 data sets. We computed average values of different performance measures such as TPR, TNR, FPR, FNR, MER, FDR and AUC based on 20 and 60 estimated DE genes by five methods (T-test, SAM, LIMMA, PCout and Proposed) for each of 100 data sets. Fig 1a and 1b represent the ROC curve based on 20 estimated DE genes by four methods for both small-and-large-sample cases, respectively. We observe that the proposed method performed better than other four methods for small-sample case (Fig 1a). On the other hand, for large-sample case (Fig 1b) proposed method keeps almost equal performance with other four methods. Fig 2 shows the boxplot of AUC values based on 100 simulated data set estimated by each of the four methods both for small-and-large-sample cases, respectively. Fig 2a and 2b represent the boxplots of AUC values with pDEG = 0.02 and 0.06, respectively. From these boxplots we obtained similar results like ROC curve for every pDEG values. We also notice that the performance of the methods increases when we increase the value of pDEG 0.02 to 0.06. Furthermore, we calculate the average values of different performance measures such as TPR, TNR, FPR, FNR, MER, FDR and AUC based on 20 (pDEG = 0.02) and 60 (pDEG = 0.06) to estimate DE genes by each of the methods. The results are summarized in Table 1. In this table the results without and within the brackets indicate average of different performance measures estimated by different methods for small-and-large sample cases, respectively. We also find the similar interpretations like ROC curve and boxplots (Table 1).

**Table 1:** Performance evaluation of different methods based on simulated gene expression dataset generated from normal distribution.

**Methods**	**With proportion of DE gene (pDEG) = 0.02**						
	**TPR**	**TNR**	**FPR**	**FNR**	**MER**	**FDR**	**AUC**
T-test	0.702 (0.932)	0.006 (0.001)	0.994 (0.999)	0.298 (0.068)	0.012 (0.003)	0.298 (0.068)	0.702 (0.932)
SAM	0.775 (0.935)	0.005 (0.001)	0.995 (0.999)	0.225 (0.065)	0.009 (0.003)	0.225 (0.065)	0.775 (0.935)
LIMMA	0.810 (0.935)	0.004 (0.001)	0.996 (0.999)	0.190 (0.065)	0.008 (0.003)	0.190 (0.065)	0.810 (0.935)
PCOut	0.838 (0.928)	0.003 (0.001)	0.997 (0.999)	0.162 (0.072)	0.006 (0.003)	0.162 (0.927)	0.837 (0.185)
Proposed	0.890 (0.935)	0.002 (0.001)	0.998 (0.999)	0.110 (0.050)	0.004 (0.002)	0.110 (0.050)	0.890 (0.950)
**Methods**	**With proportion of DE gene (pDEG) = 0.06**						
	**TPR**	**TNR**	**FPR**	**FNR**	**MER**	**FDR**	**AUC**
T-test	0.772 (0.933)	0.012 (0.004)	0.988 (0.996)	0.228 (0.067)	0.023 (0.007)	0.228 (0.067)	0.771 (0.933)
SAM	0.810 (0.933)	0.010 (0.004)	0.990 (0.996)	0.190 (0.067)	0.019 (0.007)	0.190 (0.067)	0.809 (0.933)
IMMA	0.823 (0.933)	0.009 (0.004)	0.991 (0.996)	0.177 (0.067)	0.018 (0.007)	0.177 (0.067)	0.823 (0.933)
PCout	0.837 (0.914)	0.009 (0.005)	0.991 (0.995)	0.163 (0.009)	0.016 (0.009)	0.163 (0.914)	0.837 (0.183)
Proposed	0.911 (0.959)	0.005 (0.002)	0.995 (0.996)	0.089 (0.041)	0.009 (0.004)	0.089 (0.041)	0.911 (0.933)

### Simulated gene expression profiles generated from t- distribution

**Fig 3:**
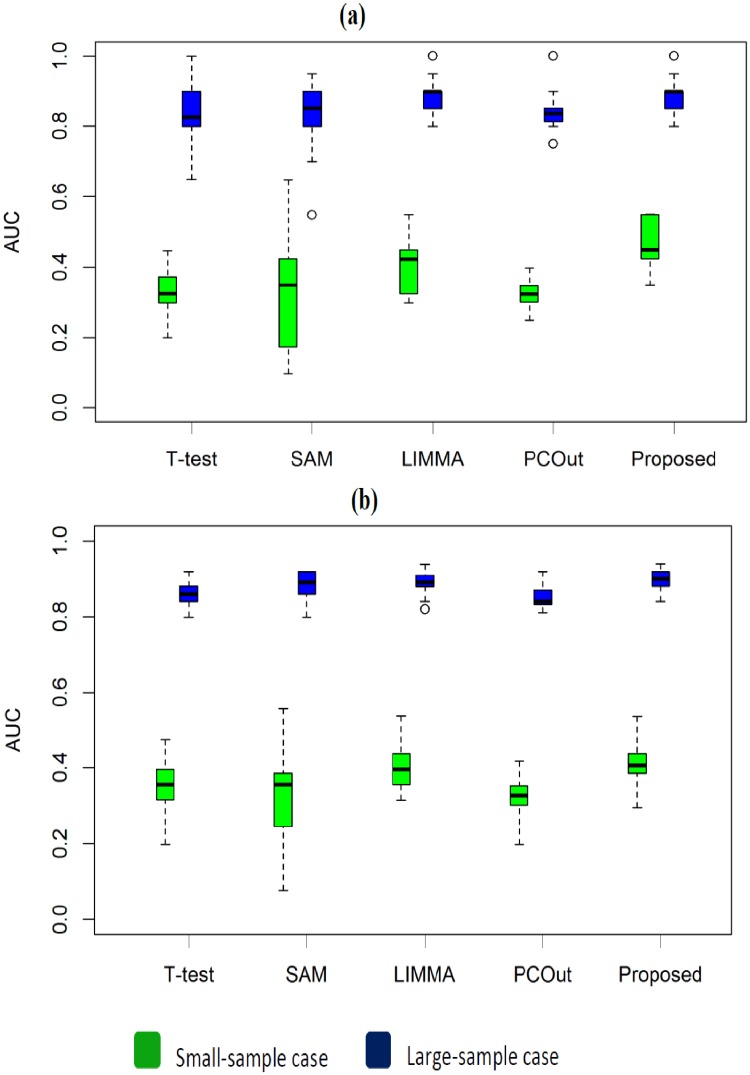
Performance evaluation using boxplot of AUC values produced by the four methods (T-test, SAM, LIMMA, PCout, and Proposed) based on 100 data sets were taken from t-distribution distribution for small-and large-sample cases (a) Boxplot of AUC values with proportion of DE gene = 0.02. (b) Boxplot of AUC values with proportion of DE gene = 0.06. Each data set contains G = 1000 genes.

We also investigated the performance of the proposed method in a comparison of other four methods for non-normal case. Accordingly we generated 100 simulated data sets from t-distribution with 10 degrees of freedom. We set the mean and variance as previously mentioned. We estimated different performance measures such as TPR, TNR, FPR, FNR, MER, FDR and AUC based on 20 estimated DE genes by four methods for each of 100 data sets. The average values of performance measures are summarized in Table 2. From this table we mentioned that the performances of all the methods become progressively worse when the datasets came from t-distribution. We also observed that the proposed method performed better than the other four methods. For example, the proposed method produces AUC = 0.469 (0.887) which is larger than 0.316 (0.830), 0.326 (0.832), 0.411 (0.880) and 0.316 (0.830) for the competitors T-test, SAM, LIMMA and PCout, respectively. The boxplots in Fig 3 and ROC curve in Fig 1(c) and 1(d) also revealed similar results like Table 2. We also noticed from boxplots that the proposed method has less variability among the other four methods. From this analysis we may conclude that the performance of the proposed method has improved than the four well-known gene selection methods.

**Table 2:** Performance evaluation of different methods based on simulated gene expression data set generated from t-distribution.

Methods	With proportion of DE gene (pDEG) = 0.02						
	TPR	TNR	FPR	FNR	MER	FDR	AUC
T-test	0.318 (0.830)	0.014 (0.003)	0.986 (0.997)	0.682 (0.170)	0.027 (0.007)	0.682 (0.170)	0.316 (0.830)
SAM	0.328 (0.832)	0.014 (0.003)	0.986 (0.997)	0.672 (0.168)	0.027 (0.007)	0.672 (0.168)	0.326 (0.832)
LIMMA	0.412 (0.880)	0.012 (0.002)	0.988 (0.998)	0.588 (0.120)	0.024 (0.005)	0.588 (0.120)	0.411 (0.880)
PCout	0.318 (0.830)	0.014 (0.003)	0.986 (0.997)	0.682 (0.170)	0.027 (0.007)	0.682 (0.166)	0.316 (0.830)
Proposed	0.470 (0.888)	0.011 (0.002)	0.988 (0.998)	0.530 (0.112)	0.021 (0.004)	0.530 (0.112)	0.469 (0.887)

### Application to colon cancer microarray data

**Fig 4:**
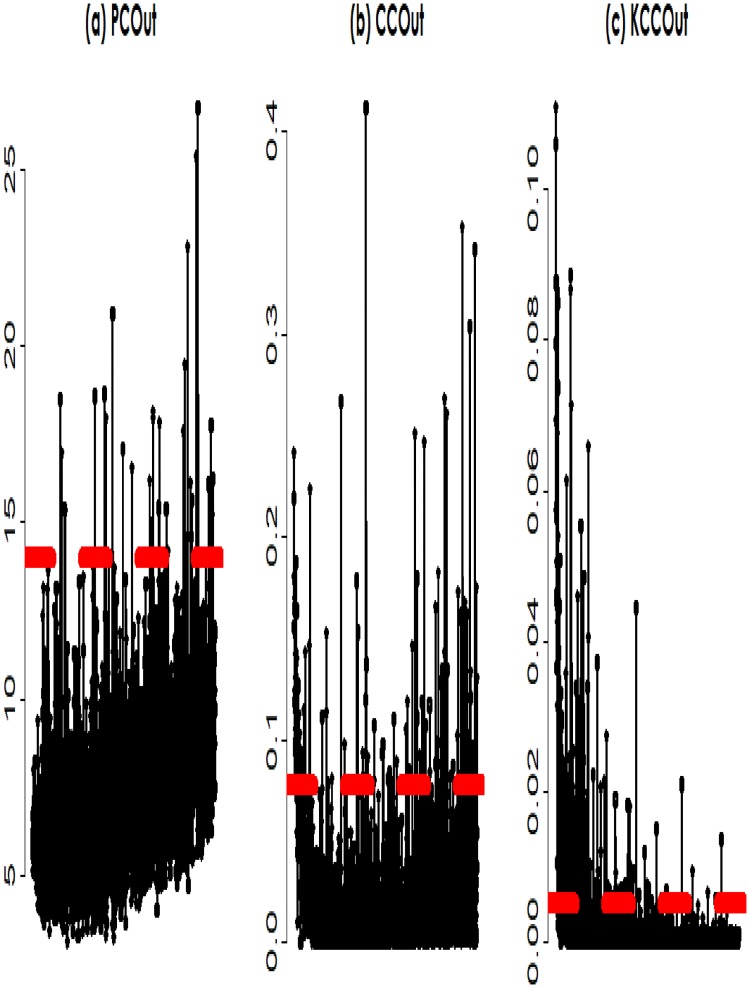
The influence value of genes using three methods: The principal components analysis (PCOut), the linear canonical correlation analysis (LCCOut), and the kernel canonical correlation analysis (KCCOut).

**Fig 5:**
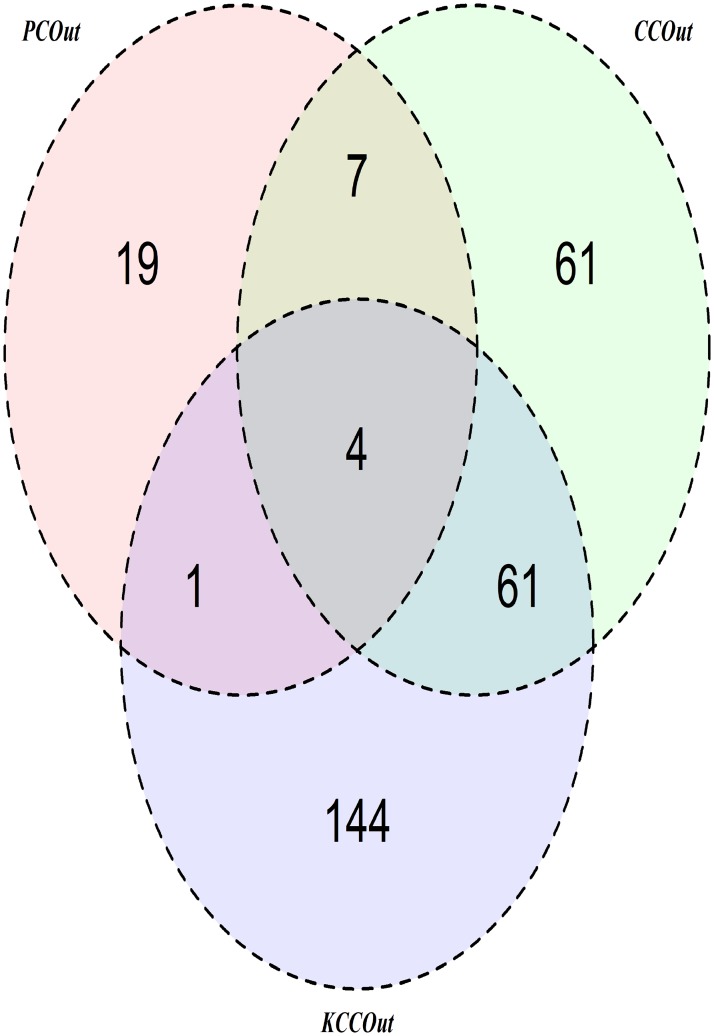
The Venn diagram of the selected genes using three methods: The principal components analysis (PCOut), the linear canonical correlation analysis (LCCOut), and the kernel canonical correlation analysis (KCCOut).

The data consists of expression levels of 2000 genes obtained from a microarray study on 62 colon tissue samples collected from colon-cancer patients [33]. Among the 62 colon tissues, tumor tissues (40) and normal tissues (22) were coded by 2 and 1, respectively. The goal here is to characterize the underlying interactions between genetic markers for their association with the colon-cancer patients and the healthy persons. In simulation studies, we observed that the multivariate approaches (the PCOut and the proposed (KCCOut)) performed better than univariate approaches. In addition to PCOut and KCCOut, we considered liner CCA (CCOut) to colon cancer data analysis. To calculate the influence value of each gene, we used these three methods, respectively. Fig 4. visualizes the plots of absolute influence value for 2000 genes. By the outlier detection technique in the one dimensional influence value of each method, we obtained 31, 133 and 210 genes using the PCOut, the CCOut and the KCCOut, respectively. To compare the selected genes, we made a Venn-diagram of the selected genes from the three methods. Fig 5. presents the Venn-diagram of the PCOut, LCCAOut, and KCCAOut methods. From this figure, we observed that the disjointedly selected genes of PCOut, LCCAOut, and KCCAOut are 19, 61, and 144, respectively. The number of common genes between PCOut and LCCAOut, and PCOut and KCCAOut, and LCCAOut and KCCAOut were 7, 1, and 61, respectively. All methods selected 4 common genes: J00231, T57780, M94132 and M87789.

**Fig 6:**
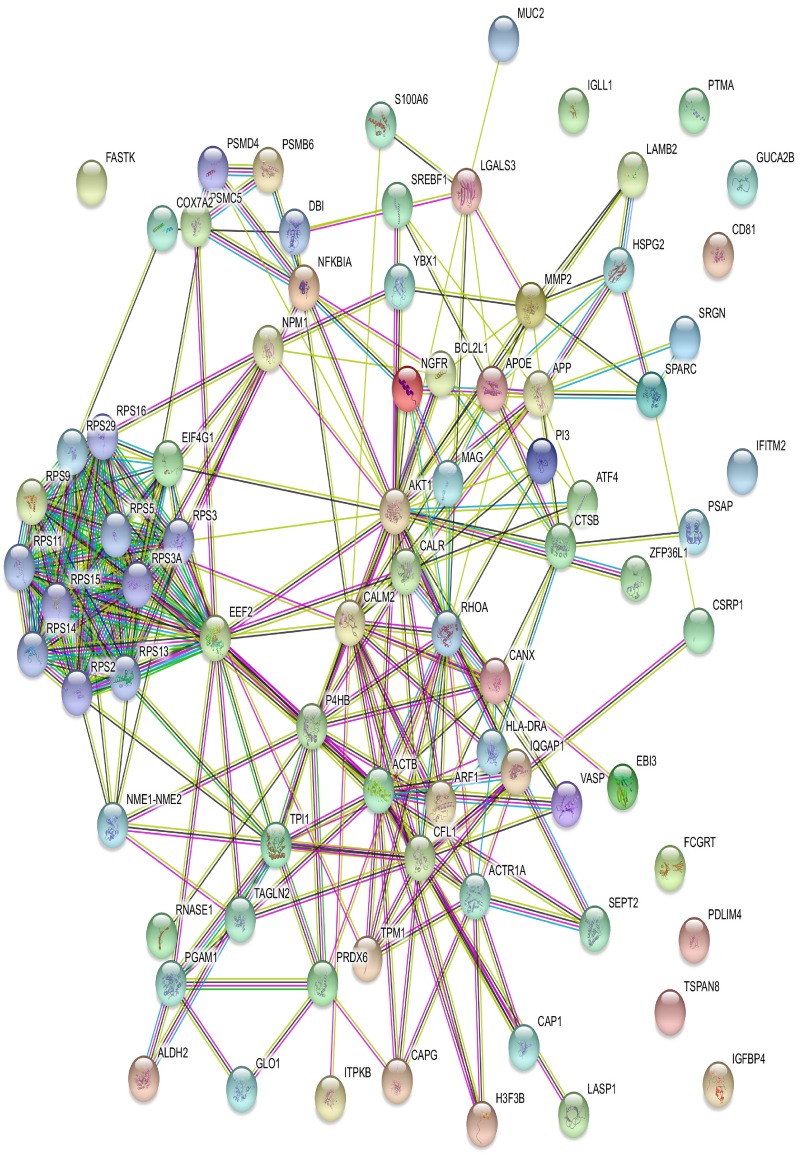
The network of the selected genes by the proposed method of colon cancer microarray data.

Genes do not function alone; rather, they interact with each other. When genes share a similar set of gene ontology (GO), they are more likely to be involved with similar biological mechanisms. To verify this, we extracted the GO of biological process categories and Kyoto Encyclopedia of Genes and Genomes (KEGG) pathway annotations of 210 genes detected by proposed KCCA using Database for Annotation, Visualization and Integrated Discovery (DAVID) [35]. The GO analysis revealed that most of genes are significantly enriched in biological adhesion, cell adhesion, viral process, multi-organism cellular process, regulation of cellular amide metabolic process etc. (see supplementary S1 Table). Table 3 presents the KEGG pathway analysis. From the table, we found that these genes are mostly enriched in toxoplasmosis, antigen processing and presentation, proteoglycans in cancer, neurotrophin signaling pathway, small cell lung cancer etc. (also see supplementary S2 Table). We also constructed the gene-gene interaction networks using STRING [36]. The STRING imports protein association knowledge from databases of both physical interactions and curated biological pathways. In STRING, the simple interaction unit is the functional relationship between two proteins or genes that can contribute to a common biological purpose. Fig 6. shows the gene-gene network based on the protein interactions among the selected 210 genes. In this figure, the color saturation of the edges represents the confidence score of a functional association. Further network analysis shows that the number of nodes, number of edges, average node degree, clustering coefficient, *p*-values are 75, 214, 5.71, 0.473 for *p* ≤ 8.22 × 10^−15^, respectively. This network of genes has significantly more interactions than expected, which indicates that they may function in a concerted effort.

**Table 3:** Top ten significant KEGG pathways for the 210 genes detected by the proposed method for Colon cancer data set.

ID	Name	No. of gene	p-vlaue
hsa05145	Toxoplasmosis	6	5.63*E* − 05
hsa04612	Antigen processing and presentation	5	7.79*E* − 05
hsa05166	HTLV-I infection	8	1.02*E* − 04
hsa04210	Apoptosis	6	1.46*E* − 04
hsa05416	Viral myocarditis	4	3.66*E* − 04
hsa04722	Neurotrophin signaling pathway	5	6.48*E* − 04
hsa05205	Proteoglycans in cancer	6	1.12*E* − 03
hsa05222	Small cell lung cancer	4	1.52*E* − 03
hsa04145	Phagosome	5	1.91*E* − 03
hsa05164	Influenza A	5	3.34*E* − 03

The proposed method can be applied to the study of other disease process, where two view data is a common task. To confirm, we have applied the proposed method to another real data set: RNA-sequence study for osteoporosis risk (Source: Tulane Center of Bioinformaties and Genomics). The details of the data and the results are provided in supplementary material, S1 File.

In addition, the data set was used to classify the colon cancer patients from the healthy controls via the PCOut and the proposed feature extraction techniques (CCOut and KCCOut) and followed by the two classifiers (the k-nearest neighbors (KNN) and liner support vector machine (SVM)). For the proposed approach, we considered the features 31, 133 and 210 that have influence effects using the PCout, the CCOut and the KCCOut, respectively. The PCOut, CCOut, and KCCOut serve as a feature extraction tool based on which the classifier is used to separate patients from healthy controls. Table 4 presents the classification error using cross-validation (2−fold and 5−fold). From these results, it is evident that the KCCOut based classification is significantly more accurate than other methods as well as methods on all features, demonstrating that the proposed method is a better tool for feature extraction.

**Table 4:** The classification error of discriminating colon cancer patients from healthy controls with cross-validations.

Feature extraction techniques	Classifier	2-fold	5-fold
**LCCOut**	SVM	12.903 ± 6.842	6.282 ± 3.598
	KNN	22.581 ± 9.124	44.615 ± 24.687
**KCCOut**	SVM	9.678 ± 2.281	9.615 ± 4.362
	KNN	29.0323 ± 13.685	41.538 ± 20.059
**PCOut**	SVM	17.742 ± 6.843	19.231 ± 8.584
	KNN	12.903 ± 11.405	40.000 ± 19.154
**All features**	SVM	14.516 ± 4.561	12.692 ± 6.853
	KNN	33.871 ± 18.247	49.231 ± 14.391

## Discussion

Kernel based machine learning methods are vital for the biomedical data analysis. The kernel based methods provide more powerful and reproducible outputs, while the interpretation of the results remain challenging. In this paper, the influence function of the kernel CCA based gene shaving method is proposed. The performance of the proposed method was evaluated on both simulated and real data set. The extensive simulation studies show the power gained by the proposed method relative to the alternative methods. The utility of the proposed method is to further demonstrate its application to analyze cancer microarray data, e.g. colon cancer microarray data. According to the influence values, the proposed method is able to rank the influence of a gene, and the genes are identified to be highly related to disease. Using an distribution based outlier detection method, the proposed method extracts 210 genes out of 2000 genes, which are considered to have a significant impact on the patients. Incorporating biological knowledge information (e.g., GO) can provide additional evidence for the results. By conducting GO, pathway analysis, and network analysis including visualization, we find evidence that the selected genes have significant influence on the manifestation of colon cancer disease and can serve as a distinct feature for the stratification of colon cancer patients from the healthy controls. This novel method can be applicable to the study of other disease processes including cancer, where gene shaving is a common task.

## Supporting information

S1 Table GO biological process categories for 210 genes for Colon cancer data set. (XLSX)

S2 Table KEGG (whole) Pathways for 210 genes for Colon cancer data set. (XLSX)

S1 File The details of the RNA-seq data and its results. (PDF)
