# Supplementary material for: Gene shaving using a sensitivity analysis of kernel based machine learning approach, with applications to cancer data
Source: PLoS One. 2019 May 23;14(5):e0217027. doi: 10.1371/journal.pone.0217027 (PMC6532884; doi:10.1371/journal.pone.0217027)
Supplement: S1 File — (PDF) [file pone.0217027.s003.pdf]

Supplementary Material

Gene Shaving using A Sensitivity Analysis of Kernel based Machine Learning Approach, with Applications to Cancer Data

Md. Ashad Alam, Mohammd Shahjaman, Md. Ferdush Rahman, Fokhrul Hossain, Hong-Wen Deng

The proposed method can be applied to the study of any disease process, where two view data is a common task. To confirm, we applied the proposed method to another real data set: RNA-sequence study for osteoporosis risk (Source: Tulane Center of Bioinformatics and Genomics). We analyzed 128 Caucasian females ongoing Louisiana Osteoporosis Study (LOS). Patients were between 20-40 years of age, including 65 with extremely high bone mineral density (BMD) and 63 with extremely low BMD The BMD value will be ascertained by Dual Energy X-ray Absorptiometry (DXA). Collect 70 ml blood from each of the 128 subjects, which will be used for the PBMs isolation (with 60ml of blood) and for measuring serum levels of vitamin D [25(OH)D] (with 10ml of blood). The extracted RNA will be stored in a -80°C freezer, which will be used for downstream methylation and 7500 gene expression analyses to attain the goal. Figure S1 shows the resulting influence values for each gene, together with the dashed red line at 0.0269 separating outline genes (above) from non-outline genes (below).

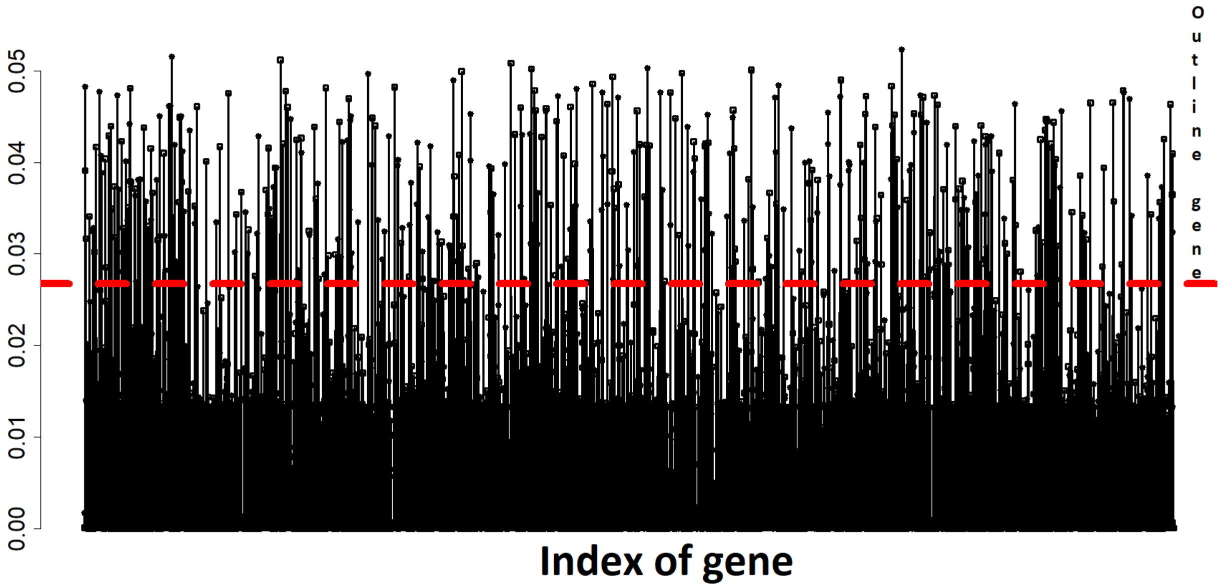

S1 Fig. Influence value of each gene: genes with vale high than the boundary 0.0269 (above dashed red line) are outline genes.

The proposed method found 423 outline genes out of 7500 genes. We conducted the gene-gene network based on protein interaction of the 423 selected genes STRING software. Figure S2 presents the network for the selected genes by the proposed method of RNA-sequence data. Further network analysis shows that the number of nodes, expected number of edges, number of edges, average node degree, clustering coefficient, PPI enrichment p-value are 405, 312, 383, 1.89, 0.255, and 0.0000572, respectively. The network has significantly more interactions than expected, indicating that these genes have more interactions among themselves than what would be expected for a random set of genes of similar sizes drawn from the genome. This finding indicates that the genes are at least biologically connected as a group. By performing gene ontology enrichment analysis, pathway analysis, gene-gene network and other studies, the proposed robust methods can find undiscovered genes in addition to significant gene pairs, which show superior performance over current ones.

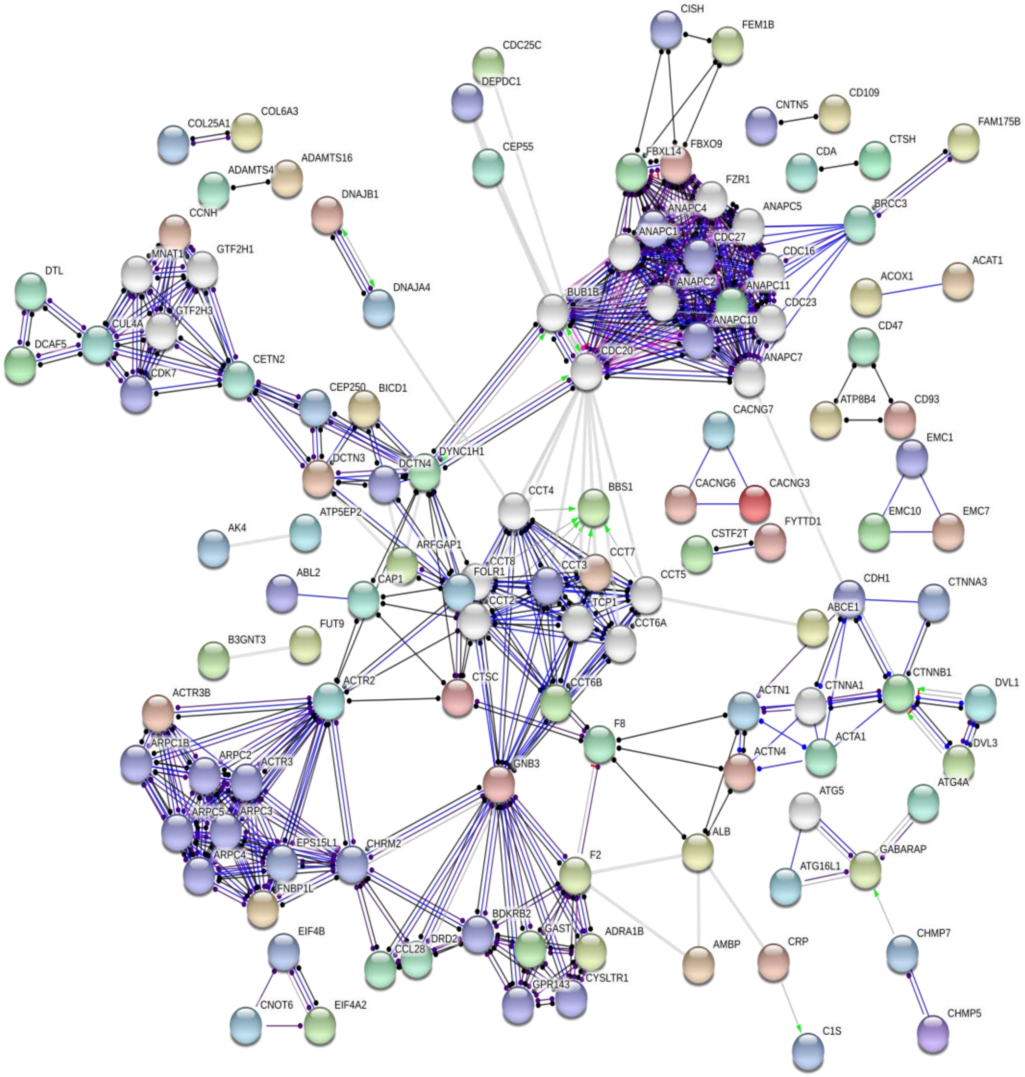

S2 Fig. The network for the selected genes by the proposed method of RNA-sequence data.
